# Discussion on effect of material on UV reflection and its disinfection with focus on Japanese Stucco for interior wall

**DOI:** 10.1038/s41598-021-01315-1

**Published:** 2021-11-08

**Authors:** Tomonori Endo, Aki Gemma, Ryoto Mitsuyoshi, Hiroki Kodama, Daiya Asaka, Midori Kono, Takeshi Mochizuki, Hiromi Kojima, Takeo Iwamoto, Saburo Saito

**Affiliations:** 1 Department of Otorhinolaryngology, Federation of National Public Service Personnel Mutual Aid Associations, Tokyo Kyosai Hospital, 2-3-8 Nakameguro, Meguro-ku, Tokyo, 153-8934 Japan; 2 Department of Otorhinolaryngology, The Jikei University School of Medicine, Tokyo, Japan; 3 Department of Laboratory Medicine, The Jikei University School of Medicine, Tokyo, Japan; 4 Eco Revival Co., Ltd., Saitama, Japan; 5 Core Research Facilities for Basic Science, The Jikei University School of Medicine, Tokyo, Japan; 6 Division of Environment Allergy, The Jikei University School of Medicine, Tokyo, Japan

**Keywords:** Infectious diseases, Public health, Civil engineering, Materials science, Optics and photonics

## Abstract

Research has previously shown that ultraviolet light C (UV-C) can inactivate unexpected infection. However, this type of potential disinfection is dramatically reduced for the shadow area such as under desk or medical equipment. Because the UV-C reflectance ratio is low on the general wall surfaces. We compared Stucco against the other materials to investigate whether we could improve disinfection for the shadow area. The reflectance ratios of UV-C irradiation of each material were examined, with particular attention to the rates for the author’s Modified Stucco. To evaluate the disinfection effects of the UV-C reflective lighting, colonies of *E. coli* and of *Staphylococcus hominis* were cultured in an agar media and counted over a certain time period after applying UV-C irradiation from a sterilizing lamp onto the investigation materials. The author’s Modified Stucco, produced reflectance ratios that was 11 times that of white wallpaper. This demonstrated that the UV-C reflected on the Stucco wall having optimum components and their compositions inhibited the number of *E. coli* and *S. hominis*, resulting in significantly disinfection effects on white wallpapers. The space with Modified Stucco and then irradiated by a UV-C may give a strong disinfection effect.

## Introduction

In recent years, many microbes have developed increased antimicrobial drug resistance^1^. It has also been discovered that these antibiotic-resistant infectious bacteria can be spread through contact with medical devices and environmental surfaces, becoming one of the causes for Healthcare Associated Infection (HAI)^2,3^. Therefore, measures to inhibit such infectious spread are urgently needed. One infection control strategy involves the inactivation of infectious bacteria using an ultraviolet (UV) germicidal lamp^3,4^. UV lights below 400 nm, especially around 254 nm (UV-C) show strong microbe inactivation effects by being absorbed into the bacteria DNA and inhibiting DNA from replicating^5^. However, the UV-C light is substantially absorbed into standard wall surfaces, reducing the UV-C light reflecting off of the wall surface^33^. In other words, UV-C does not reach areas that are shaded under desks or medical equipment. This reduces the overall germicidal effect of the UV-C treatment compared to the strong disinfection effect of direct UV-C irradiation onto bacteria samples.

In this study, we investigated whether Stucco, a traditional Japanese inner wall material, could be used as an effective disinfection if UV-C lighting could be projected onto the wall surfaces and reflected back into the examination room environment. For more than 1300 years, Japanese construction practices have used mortar as a base coating for outer walls and Stucco for finishing inner walls^6^. A typical example is the historic use of Stucco for outer walls is in Japanese castles of the 1600s^7^. The main ingredient in Stucco is Ca(OH)_2_, prepared burning calcium hydroxide mined from limestone and distilled at the super high temperature of 900 ℃ or higher. In the past, fillers, such seaweed and fibers as were added, but in the present polymers are added as thickening agents. Most Stucco formulas are a mixture of natural materials. When the Stucco is applied to the wall surface, it begins to harden by absorbing CO_2_ from the air through the chemical reaction Ca(OH)_2_ + CO_2_ → CaCO_3_ + H_2_O. The Stucco continues to harden for at least 10 years, eventually becoming calcium carbonate (CaCO_3_), a high-duty, limestone-like hard surface. For this reason, Stucco has historically been used for roof tiles and for both outer and inners walls requiring high strength^8,9^.

However, in the 1960s the Japanese economy grew rapidly and the use of Stucco in Japanese building practices fell due to a new dry construction method, which shortened the construction period, allowing for mass production and reduced costs. This construction trend changed Japanese inner wall materials from Stucco to white wallpaper or white paint. The use of Stucco decreased because the longer construction time and finishing time, which depended upon the techniques of Stucco artisans^6^. At present, the use of Stucco is mainly for special locations, such as in private homes and in hotels as wall art. The Stucco is a traditional material for inner walls in Japan as well as in foreign countries.

In recent years, the use of Stucco for interior walls has received renewed attention. The Stucco used in historical buildings is not only artistic, but also has the advantage of strong wall strength and long service life. Recent studies have shown that Stucco walls absorb and release moisture from the atmosphere, thereby preventing rapid changes in room humidity^10,11^. Humidity control is the most important strategy to reduce indoor fungal growth. A room with Stucco walls is reported to have a high anti-fungal effect due to humidity control^12^. Powder lime, with its strong alkaline properties, is used in many countries for the prevention and control of avian influenza virus^13^. Similarly, fine-grained lime Stucco walls have an antimicrobial activity under moist conditions. In contrast, grain wall materials with a surface of porous adsorbs bacteria and have no antimicrobial effect^14^. This antimicrobial effect of fine-grained Stucco walls would allow to be used in health care unit. Stucco walls also efficiently absorbs volatile organic compounds (VOCs) generated during construction associated sick building syndrome^15^. These functionalities of the Stucco are due to the porous mediums structure such as carbon-based adsorbent. In addition, Stucco is made of inorganic materials, so it has excellent fire resistance^16^. Stucco has the potential to make residence more comfortable and safer than conventional wallpaper or paint.

Through this study of UV-C light irradiated onto a Stucco surface and the resulting reflected UV-C light waves released by the irradiated Stucco, it was shown that Stucco has a high UV-C reflectance ratio and that this reflected light has a high disinfection effect, compared to white wall papers and paints which are used for a general inner wall. In the hopes of increasing the reflectance ratio, this paper’s authors altered conventional Stucco by creating a unique formula, thereby creating a Modified Stucco. This modified version of Japanese Stucco was compared favorably to the reflectance ratio of aluminum (aluminum foil), a material previously reported as having the highest UV-C reflectance ratio^17^.

## Methods

### Powder materials and average diameters of their particles

The powder materials used for preparing the Modified Japanese Stucco, with the authors’ unique composition and particle diameters, are as follows: calcium hydroxide (a generous gift from Nippon Plaster Co., Ltd. Tochigi, Japan), calcium carbonate in three different particle sizes (a generous gift from Nippon Plaster Co., Ltd. Tochigi, Japan), calcium hydroxide (Esukaron #2300, Sankyo Seifun Co., Ltd. Okayama, Japan), barium sulfate (022-00425, Fujifilm Wako Pure Chemical Co., Ltd. Osaka, Japan), barium sulfate (W-6, W-1, P-30, Takehara Kagaku Kogyo Co., Ltd. Hyogo, Japan).

The diameters for the particles of the three types of calcium carbonate were provided by the maker. Those values were measured by a laser diffraction particle size analyzer (SALD-200V, Shimadzu Co., Ltd. Kyoto, Japan).

The average diameters of the particles of microparticulate calcium carbonate and magnesium sulfate were measured by dynamic light scattering and zeta potential measurement systems (Zetasizer Nano ZS, Malvern Panalytical Ltd. Worcestershire, United Kingdom).

### UV-C reflectance ratios and irradiance of each material

Using a UV–visible–Near InfraRed (UV–Vis–NIR) spectrophotometer (UV-3600, Shimazu Co., Ltd. Kyoto, Japan), a source light was dispersed into monochrome light in the UV-C wavelengths through diffraction grating. The resulting UV to visible light (240–780 nm) irradiated the sample materials. The light reflected back from the surface of the sample materials was measured using the integrating sphere attachment of the spectrophotometer and the reflectance ratios were calculated. The UV-C reflectance ratios of Stucco with different mixing ratios and particle sizes of each material was measured.

By changing formulation and particle size of CaCO_3_ and BaSO_4_, we created a sample of Stucco using powder materials and thickener. Water was added to these material at room temperature to make a paste, which was applied to the plasterboard by an artisan. Based on these reflectance data, the Stucco was modified to improve the UV-C reflectance. The Modified Japanese Stucco prepared by this paper’s authors was based on JIS A 6919 Japanese Industrial Standards (JIS) for Stucco^18^ (“Supplementary data”).

The irradiated materials on which UV-C reflectance ratios were measured are follows: aluminum foil (MY FOIL, UACJ Foil Co., Ltd. Tokyo, Japan), aluminum plate (N96620, Nippon Light Metal Co., Ltd. Tokyo, Japan), commercial Japanese Stucco (Shikkui Kurumu Uchi^®^, Nippon Plaster Co., Ltd. Tochigi, Japan), white wallpaper (FE6716, Sangetsu Co., Ltd. Aichi, Japan), white paint (Suisei-Wide V, Nippe Home Products Co., Ltd. Tokyo, Japan), and the Modified Japanese Stucco blended by the authors. The other metals examined were stainless steel plate (SUS304 2B, Nippon Steel Stainless Steel Co., Ltd. Tokyo, Japan), and copper plate (C1100P, Mitsubishi Materials Corporation Co., Ltd. Tokyo, Japan). In addition, common Japanese cedar lumber, mortar and concrete were measured.

Using a UV irradiance meter (UV-M02, Orc Manufacturing Co., Ltd. Tokyo, Japan), UV-C irradiance at the agar media after passing through various test boxes was also measured. The test boxes in which UV-C irradiance was measured are follows: aluminum foil (back side, matt), authors’ Modified Stucco, commercial Stucco, and white wall paper.

### The bacteria used for measuring the disinfection effects

The bacteria used *Escherichia coli* (*E. coli*; JM109 strain, Takara Bio Inc. Shiga, Japan), and *Staphylococcus hominis* (*S. hominis*). These are Gram-negative and Gram-positive bacteria with different bacterial cell wall structures. Instead of the pathogenic microorganisms that are problematic in hospitals, these bacteria were used as surrogate microorganisms for this study. *S. hominis* were collected by placing the standard agar media at 0.8 m height from the floor for 15 min. with careful aseptic handling, and by passive microbial sampling. The collected bacteria were cultured at 37 ℃ for 48 h. From the various cultured bacteria, *S. hominis* were identified by MALDI-TOF MS (autoflex speed™ MALDI-TOF/TOF MS, Bruker Corp. Massachusetts, USA).

### Disinfection effects of UV-C reflective lighting

**Figure 1:**
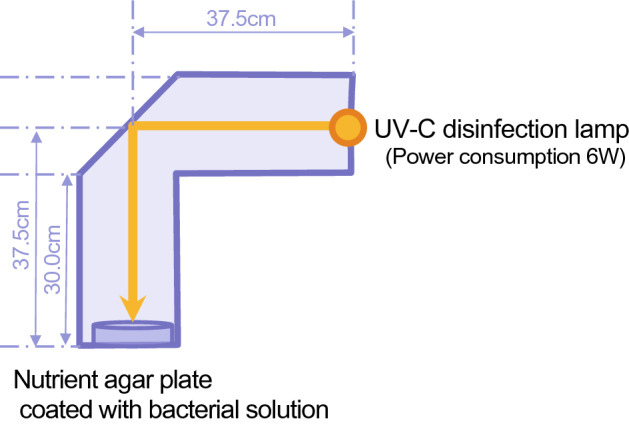
Test apparatus for evaluating the disinfection effects of UV-C reflected irradiation to various materials. UV-C from a UV germicidal lamp (6 W, 254 nm) was reflected in a 90° on the inner wall covered with various materials. The reflected lights were irradiated on the agar plate coated with bacteria solution.

The disinfection effects of UV-C irradiation reflected off of each wall material were evaluated using a test wall, which simulated an inner wall (Fig. 1). These wall sections were uniformly coated or covered with each material. A light wave reflected 90° from the direct light wave of a UV-C germicidal lamp (power consumption 6 W, UV irradiance 19 μW cm^−2^, 254 nm, Toshiba Lighting & Technology Co., Ltd. Kanagawa, Japan) irradiated the standard agar media (Trypto-Soya Agar, Petri dish diameter: 8 cm) which had been applied with either the *E. coli* or *S. hominis*. Then, the number of colonies that had cultured at 37 ℃ for 48 h. was counted to compare the disinfection effects.

The materials used in this portion of the study were commercial Stucco, the Stucco with the formula created by the authors, and the aluminum foil. White wallpaper was used as a reference. The number of bacteria was prepared solution concentration (*E. coli*; 3.47 × 10^6^ CFU/ml, *S. hominis*; 3.93 × 10^6^ CFU/ml) and this solution was applied onto the standard method agar with 50 μl.

### Statistical analysis

Statistical analyses for disinfection effects of UV-C reflected irradiation were carried out using GraphPad Prism 9.1.0 software (GraphPad Software, Inc., San Diego, CA, USA). The quantitative data (n = 3) for disinfection are presented as mean ± standard deviation. The evaluation of the bactericidal effect of UV-C reflected lighting was analyzed by two-way ANOVA. The significance of the results of the analysis was defined as p < 0.05.

## Results

### Average diameters of CaCO_3_ and BaSO_4_ particles in powder

Since we used several different sizes of calcium carbonate and barium sulfate particles, we measured the average diameter of these particle sizes (ps). The average diameters of the particles in CaCO_3_ measured by particle size analyzer were 8.9 μm, 6.0 μm, 3.0 μm, and 1.0 μm, and described as CaCO_3_ (ps = 8.9 μm), CaCO_3_ (ps = 6.0 μm), CaCO_3_ (ps = 3.0 μm), CaCO_3_ (ps = 1.0 μm).

The average diameters of the particles in barium sulfate were determined as 4.8 μm, 1.7 μm, 1.1 μm, 0.3 μm, and described as BaSO_4_ (ps = 4.8 μm), BaSO_4_ (ps = 1.7 μm), BaSO_4_ (ps = 1.1 μm), BaSO_4_ (ps = 0.3 μm).

### UV reflectance ratios for each material

**Figure 2:**
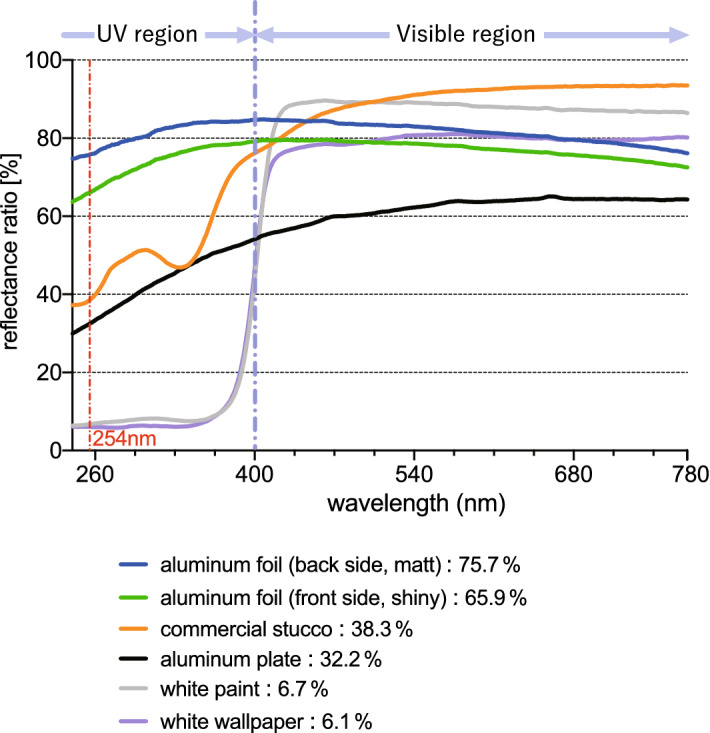
Reflectance ratios of various materials by UV and visible lights using UV–Vis–NIR spectrophotometer.

As shown in Fig. 2, the reflectance ratios of UV-C (254 nm) light for the aluminum foil (back side, matt), the aluminum foil (front side, shiny), the Japanese commercial Stucco, the aluminum plate, the white paint and the white wallpapers were, 75.7%, 65.9%, 38.3%, 32.2%, 6.7%, and 6.1%, respectively. The ratios of the stainless steel plate, copper plate, Japanese cedar lumber, mortar, and concrete were 24.5%, 26.4%, 4.7%, 21.2%, and 14.9%, respectively (Supplementary Fig. S1). Among the materials measured in this study, the aluminum foil (backside, matt) showed the highest reflectance ratio.

### Changes in UV-C reflectance ratios and irradiance of Stucco by changing composition ratio and material

**Figure 3:**
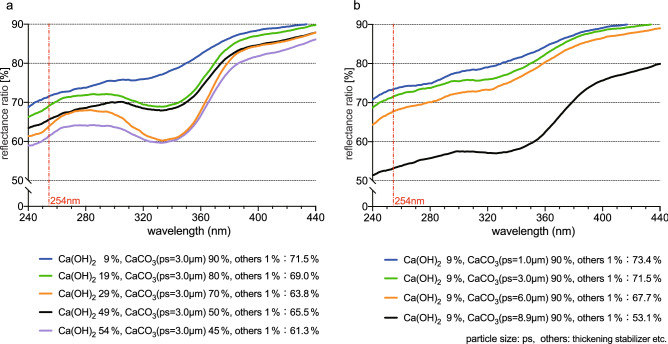
Reflectance ratios of UV lights by (**a**) changing amounts and (**b**) particle diameters of CaCO_3_.

**Figure 4:**
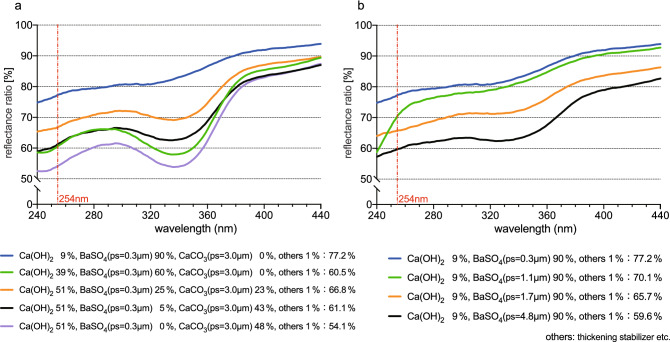
Reflectance ratios of UV lights by replacing CaCO_3_ with BaSO_4_, (**a**) changed in weight % and (**b**) particle diameter of BaSO_4_.

To increase the reflectance ratio of the commercial Stucco, the composition and the average particle diameters were changed. Standard commercial Stucco has Ca(OH)_2_ as a main component, CaCO_3_ as the second highest ingredient, and smaller amounts of sand, fibers, and a thickening agent as miscellaneous ingredients. The basic components in the Stucco used in this study are mainly Ca(OH)_2_, CaCO_3_ and a thickening agent. The UV-C reflectance ratios produced by changing the amount (%) of CaCO_3_ are shown in Fig. 3a. The changes of the ratios produced by changing the average diameters of CaCO_3_ particles at the fixed amount ratio (%) of CaCO_3_ and Ca(OH)_2_ are shown in Fig. 3b. The changes in ratios produced by replacing CaCO_3_ with BaSO_4_ were also examined (Fig. 4).

By increasing the amount (weight %) of CaCO_3_ by 45%, 50%, 70%, 80%, and 90%, the UV-C (254 nm) reflectance ratios were 61.3%, 65.5%, 63.8%, 69.0%, and 71.5%, respectively (Fig. 3a). By changing the average diameters of the CaCO_3_ particles to 8.9 μm, 6.0 μm, 3.0 μm, and 1.0 μm, the respective reflectance ratios were 53.1%, 67.7%, 71.5%, and 73.4%, respectively (Fig. 3b).

By increasing the amount (weight %) of BaSO_4_ from 0 to 5%, 25%, 60%, and 90%, the reflectance ratios of UV-C (254 nm) showed higher from 54.1 to 61.1%, 66.8%, 60.5%, and 77.2%, respectively (Fig. 4a). By changing the average diameters of the BaSO_4_ particles to 4.8 μm, 1.7 μm, 1.1 μm, 0.3 μm, the reflectance ratios were 59.6%, 65.7%, 70.1%, 77.2%, respectively (Fig. 4b).

**Figure 5:**
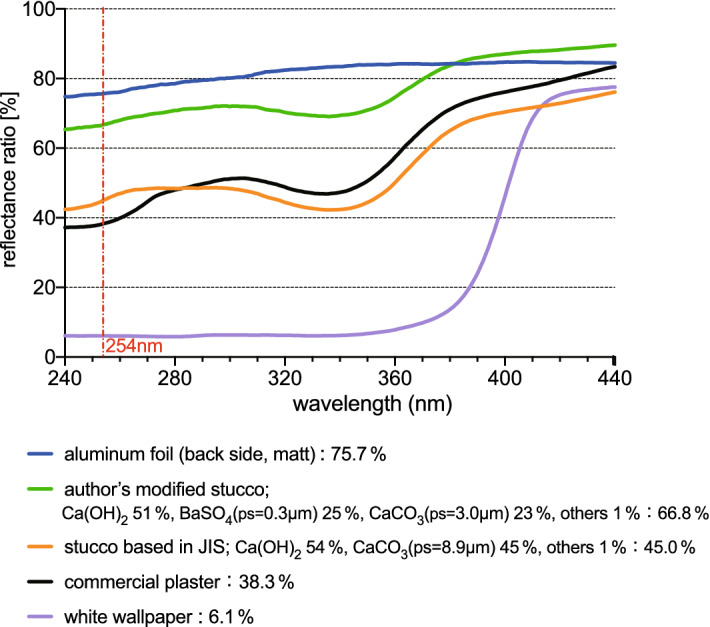
Reflectance ratios of UV lights for various materials and the author’s created Stucco.

According to JIS A 6919, the amount of Ca(OH)_2_ should amount to more than a half of the total Stucco. In the Stucco composition based on Standards in Japan, JIS A 6919 (Ca(OH)_2_ 54%, CaCO_3_ (ps = 8.9 μm) 45%, with other ingredients, including a 1% thickening agent), the UV-C (254 nm) reflectance ratio was 45.0%, showing a rate close to the commercial Stucco. In addition, in the authors’ Modified Stucco composition, which we determined based on the characteristics of CaCO_3_ and BaSO_4_ stated above, that is, Ca(OH)_2_ 51%, BaSO_4_ (ps = 0.3 μm) 25%, CaCO_3_ (ps = 3.0 μm) 23% and 1% thickener, the UV-C reflectance ratio rose to 66.8% (Fig. 5).

Next, UV-C irradiance at a distance of 75 cm from the direct UV-C lamp was 30 μW cm^−2^. In contrast, UV-C irradiance at the agar media after passing through a test box with aluminum foil (back side, matt), authors’ Modified Stucco, commercial Stucco, and white wall paper was 148 μW cm^−2^, 21 μW cm^−2^, 6 μW cm^−2^, and 1 μW cm^−2^, respectively.

### Disinfection effects of UV-C reflected irradiation

The log reduction of *E. coli* and *S. hominis* in direct UV-C light was evaluated. The UV dose (Irradiance × exposure time) required to 5.928-log_10_ reduction of *E. coli* was 3.6 mJ cm^−2^. The UV dose required to 4.254-log_10_ reduction of *S. hominis* was 18 mJ cm^−2^ (Supplementary Fig. S2).

**Figure 6:**
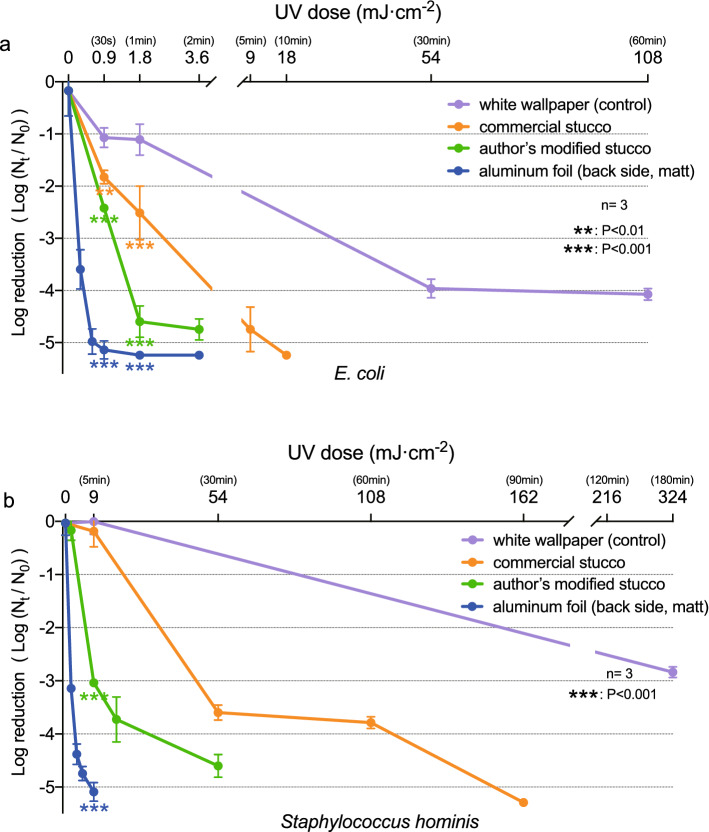
Log reduction (Log (N_t_/N_0_)) vs UV dose of UV-C reflected off various materials: the UV-C was irradiated to standard agar media applied with (**a**) *E. coli* and (**b**) *S. hominis*. UV dose is the UV intensity at a distance of 75 cm from the direct UV-C lamp (6 W, 254 nm). Data are presented as mean ± standard deviation. **p < 0.01, ***p < 0.001.

As shown in Fig. 6a, 1.11-log_10_ reduction of *E. coli* were found after 1 min of irradiation time onto the white wallpaper (the common inner wall material) and 3.96-log_10_ reduction at 30 min. In contrast, we observed log reduction of *E. coli* at only 30 s of irradiation for the commercial Stucco, the aluminum foil and the authors’ Modified Stucco (composed of Ca(OH)_2_ 51%, BaSO_4_ (ps = 0.3 μm) 25%, CaCO_3_ (ps = 3.0 μm) 23% and thickening stabilizer etc. 1%). We observed 4.6-log_10_ reduction and 5.24-log_10_ reduction, at 1 min for the authors’ modified Stucco and the aluminum foil, showing the significant difference in disinfection effects compared to white wall paper (p < 0.001, p < 0.001, respectively).

In the same experiment using the *S. hominis*, we observed more than 4-log reduction at 1 min for the aluminum foil, at 30 min for the originally created Stucco, at 90 min for the commercial Stucco. In contrast, we observed 2.84-log_10_ reduction at 180 min for white wallpaper. At 5 min of UV irradiation, authors’ Modified Stucco showed a significant difference in the disinfection effects compared to white wall paper (3.04-log_10_ reduction, p < 0.001; Fig. 6b).

## Discussion

There is increasing interest in the role of disinfection for multi-drug antimicrobial resistanc^19^. Also, COVID-19, the novel pandemic infectious disease has become a worldwide issue and is recently reported to become a growing menace in the future^20^. UV disinfection has been reported to be effective in reducing of multi-drug resistant organisms^21^ and COVID-19^22^. Since contamination of infectious microbes is noticeable at the bedsides and on the flooring under beds^23^, the risks posed by these microbes in healthcare environments has gained greater recognition. The infectious substances are diffused by contact with hands, transference from the floor to shoes, and the movement of people. Sterilizing the sites where the infectious microbes have spread is a basic countermeasure^24^ and is expected to effectively inactivate the infectious agents. However, exposure to UV-C light, one method of microbe inactivation, may risk impairment of the skin and the eyes. Yet, UV-C lighting is reported for its usefulness^25,26^ because it shows the inactivation of microbes with high efficiency when the lighting is used safely^27^. In recently, UV-LED lighting (ultraviolet light emitting diode) is attracting attention in the food field^28^, an area of limited available microbe inactivation techniques, as well as the medical field.

This study paid attention to Stucco, which has been a traditional Japanese wall material. Stucco walls are very pure white, make rooms bright, and their surface textures are artistic and very beautiful. Around the world, Stucco walls have had specific historical cultural uses. For example, Holy Trinity Cathedral in Chicago, with Stucco finishing on its brick construction, is known as historical architecture. This study was intended to demonstrate whether Stucco has a new value and highly effective functionality even in modern times in Japan. The aluminum wall surface (aluminum foil) showed the highest UV-C reflectance ratios among the materials measured in this study, as previously reported^17^. By analyzing the reflectance effect on experimental Stucco walls, the Stucco showed a high reflectance ratio not only in the visible region, but also notably in the UV-C region, as compared with the white wallpaper and white paint commonly used (Fig. 2). The UV-C reflectance ratio of the commercial Stucco was 1.2 times that of the aluminum plate, 5.7 times that of the white paint, and 6.3 times that of the white wallpaper. Of special note is that the Stucco gave a reflectance ratio higher than the aluminum plate (Fig. 2).

For the purpose of investigate the reflectance ratio of the wall surface coated with Stucco, the weight % or particle size of CaCO_3_ and BaSO_4_ have been changed. By increasing the amount of CaCO_3_, the UV-C reflectance ratios increased (Fig. 3a). In the same amount (weight %) of CaCO_3_, the smaller the average diameters of CaCO_3_ particles were, the more the UV reflectance ratios rose (Fig. 3b). By replacing CaCO_3_ with BaSO_4_, a natural inorganic substance, and increasing the amount (weight percent) of BaSO_4_, the trend of a rise in the ratio of reflectance was shown (Fig. 4a). When the average diameter of BaSO_4_ particles was smaller, the UV-C reflectance ratio increased (Fig. 4b). In short, CaCO_3_ which is the second main component of the Stucco formula can be used to increase the reflectance ratio by reducing the particle size and increasing its weight percent. Alternatively, the reflectance ratio of the Stucco can also be more increased by using BaSO_4_, which is a smaller particle size and increasing its weight percent, instead of CaCO_3_.

Stucco walls are almost composed of natural white inorganic materials. BaSO_4_ is also a white natural mineral material, it has high chemical stability with a decomposition temperature of 1600 ℃^29^, which is higher than CaCO_3_^30^. BaSO_4_ is safe and familiar to us because it is used in X-ray contrast media for the gastric. There would be no problem if BaSO_4_ was added to Stucco as a substitute for CaCO_3_. However, the smaller particle size of the second component may cause cracks in the wall due to long-term vibration. This problem may be solved by adding fibers to the Stucco.

In the Stucco formulated to be close to the reflectance of commercial Stucco (UV reflectance 45.0%, Fig. 5), the particle size of CaCO_3_ was 8.9 μm. Therefore, UV reflectance may be increased by using CaCO_3_ with smaller this particle size. As shown in Fig. 3b, the reflectance of CaCO_3_ (ps = 8.9 μm) is 53.1%, so the reflectance may be increased instead of any particle size of same amount BaSO_4_ (Fig. 4b). No cracks appeared on the wall within 14 days of applying BaSO_4_ (ps = 0.3 μm), while CaCO_3_ (ps = 1.0 μm) caused cracks on the wall (Supplementary Fig. S3). The authors considered these characteristics, one example of a modified Stucco was prepared by selecting more than half of Ca(OH)_2_, about a quarter of CaCO_3_ (ps = 3.0 μm) was selected to prevent cracking, and about a quarter of BaSO4 (ps = 0.3 μm) was selected to improve reflectivity.

The unique Stucco compositions reached UV-C reflectance ratios close to the level of aluminum foil (66.8%, Fig. 5). The author’s Modified Stucco produced reflectance ratios that was 11 times that of white wallpaper. These results demonstrated that, by selecting an optimum composition and particle diameter of CaCO_3_ or adding BaSO_4_, a Stucco wall surface can be used to show a very effective reflectance of UV-C light.

In addition to increasing the UV reflectance, UV-C irradiance was high in proportion to UV-C reflectance. The author's modified Stucco had 21 times UV-C irradiance of white wall paper. In short, by increasing UV-C reflectance also made it possible to increase the UV-C irradiance. Improvement in UV irradiance of Stucco wall is more effective than UV reflectance ratio. The reason for effective increase in UV irradiance may be Stucco walls caused reflections and scattering of UV in space. This effect on UV irradiance may also be expected in the room with Stucco wall.

We examined the disinfection effects against *E. coli* and *S. hominis* using the irradiation light waves of a UV-C germicidal lamp. More than 4 log reduction of *E. coli* and *S. hominis* required UV dose was 3.6 mJ cm^−2^ for *E. coli* and 18 mJ cm^−2^ for *S. hominis*. Comparing the UV resistance of *E. coli* and *S. hominis* to direct UV-C light, *S. hominis* required several times more UV dose than *E.coli* (Supplementary Fig. S2). As shown in Fig. 6, both the Stuccos with the unique compositions created by the authors and the Japanese commercial Stucco significantly decreased the time to be sterilized compared with white wallpaper.

The rapid decrease in the reflectance ratios of wallpaper and paint in the lights in UV region may be caused by the low transmittance of UV-C light through vinyl chloride^31^, a main component of wallpaper, and by the absorption of UV-C light by titanium dioxide^32^, used in the coloring of the paint. For the reasons stated above, the inactivation of infectants wanes substantially on the surface of objects irradiated by the reflection effect of UV-C light compared with direct UV-C lighting. UV-C light and wall coating materials which reflect UV-C light have been reported to reduce the inactivation time against microbes in a room^33^. Therefore, a room space which has both Stucco walls and a UV-C irradiation device are expected to reduce the time needed to inactivate infectious microbes more than other conventional ways, even against the shadows under desks and medical equipment. This information shows that this combination may be effective for inactivation of infectious substances on the space where the lights irradiated off the wall of objects or medical devices in the examination room of otolaryngology where a lot of those microbes may be found. The efficient inactivation of microbes in a short time may reduce the examination interval from one patient to the next. Since UV radiation over long intervals causes deterioration and discoloring of the resin in expensive medical devices, high radiation effects for a short time gives many benefits. Furthermore, Stucco has a history of longtime usage in many countries, so its applications are very beneficial.

This study was performed in the limited area as a model experiment in the laboratory for the inactivation test only against *E. coli*, and *S. hominis*, which are surrogate microorganisms for pathogenic microorganisms. The challenge in the near future, is necessary to verify the improvement of disinfection effect against microbiological associated with HAI such as ESKAPE (*Enterococcus faecium*, *Staphylococcus aureus*, *Klebsiella pneumoniae*, *Acinetobacter baumannii*, *Pseudomonas aeruginosa* and species of Enterobacter) pathogens^34^, is to explore Stucco components showing high UV reflection rates, apply to the deep UV-LED which came into practical use recently, and investigate infectious substances in the real medical environment.

## Conclusions

CaCO_3_ which is the second component of the Stucco can be increased the UV reflectance by increasing its weight percent with a small particle size of 3.0 μm. Alternatively, the UV reflectance of the Stucco can also be increased by using more BaSO_4_, which is a small particle size of 0.3 μm, instead of CaCO_3_. As an example of modified Stucco, selecting more than half of Ca(OH)_2_ and about a quarter of CaCO_3_ (ps = 3.0 μm), and about a quarter of BaSO_4_ (ps = 0.3 μm) can significantly improve UV reflectance.

The space coated optimized Stuccos showed an improvement of UV-C reflectance ratios close to that of aluminum foil (front surface, shine). Thus, when a UV-C light source is used in a room with Stucco walls which have been made with an optimum composition of elements, disinfection effects may be significantly obtained, not only under direct irradiation, but also from the irradiating light being reflected off the wall.

## Supplementary Information

Supplementary Information.

## Abbreviations

UV: Ultraviolet
*E. coli*: *Escherichia coli*
CFU: Colony-forming unit
HAI: Healthcare-associated infection
