# Supplementary material for: Discussion on effect of material on UV reflection and its disinfection with focus on Japanese Stucco for interior wall
Source: Sci Rep. 2021 Nov 8;11:21840. doi: 10.1038/s41598-021-01315-1 (PMC8575928; doi:10.1038/s41598-021-01315-1)
Supplement: Supplementary file 1 — Supplementary Information. [file 41598_2021_1315_MOESM1_ESM.docx]

**Supplementary Information**

**Discussion on Effect of Material on UV Reflection and Its Disinfection with Focus on Japanese Stucco for Interior Wall**

*Tomonori Endo^1,2^, Aki Gemma^1,2^, Ryoto Mitsuyoshi^2^, Hiroki Kodama^2^, Daiya Asaka^2^, Midori Kono^3^, Takeshi Mochizuki^4^, Hiromi Kojima^2^, Takeo Iwamoto^5^, Saburo Saito^6^

1. Department of Otorhinolaryngology, Federation of National Public Service Personnel Mutual Aid Associations, Tokyo Kyosai Hospital, Tokyo, Japan

E-mail: tomonori-endo@jikei.ac.jp Tel: +81-3-3712-3151

2. Department of Otorhinolaryngology, The Jikei University School of Medicine, Tokyo, Japan

3. Department of Laboratory Medicine, The Jikei University School of Medicine, Tokyo, Japan

4. Eco Revival Co.,Ltd, Saitama, Japan.

5. Core Research Facilities for Basic Science, The Jikei University School of Medicine, Tokyo, Japan.

6. Division of Environment Allergy, The Jikei University School of Medicine, Tokyo, Japan

**Summary of the definition of Japanese stucco for interior finishing in Japanese Industrial Standards(JIS) A 6919 (2020)**

In the past, stucco artisans determined their own formulations, but the definition of Japanese stucco was first established in JIS in 2020. The main components of Japanese stucco are as follows: Ca(OH)_2_ is more than 50% of the total stucco formulation. Cement or gypsum should not be used as a bonding material for stucco. Aggregates, glues, fibers, pigments, and thickeners must not affect the quality, and should be less than 6.8% of the total stucco mixture. Based on the fact that the organic material is less than 6.8%, this is a condition that indicates non-combustible properties. The stucco has a water retention rate of at least 34%. Cracking of the stucco wall should not occur for 14 days after the first application in a wind tunnel chamber under conditions of 3 m/s wind velocity. The adhesive strength of stucco to plasterboard must be at least 0.1 N/mm^2^. ****

**Supplementary Figure S1**. Reflectance ratios of various metals and wall materials by UV and visible lights using UV-Vis-NIR spectrophotometer.

**Supplementary Figure S2**. Log reduction ( Log ( N_t_ / N_0_) ) of *E.coli* and *Staphylococcus hominis* vs UV-dose (mJ·cm^-2^). UV-C lamp (6W, 254nm) from a distance of 75cm was irradiated on the agar plate coated with bacteria solution. Data are presented as mean ± standard deviation.

**Supplementary Figure S3**. Wall images of CaCO_3_ (ps=1.0μm), CaCO_3_ (ps=3.0μm), and BaSO_4_ (ps=0.3μm) applied to plasterboard. Weight percentages of CaCO_3_ and BaSO_4_ are 90%, Ca(OH)_2_ is 9%, and the thickener is 1%. Cracks appeared in the wall surface with CaCO_3_ (ps=1.0μm). No cracks were observed in the wall use of CaCO_3_ (ps=3.0μm) and BaSO_4_ (ps=0.3μm).
